# Preliminary Research: Description of Lip Print Patterns in Children and Their Parents among Deutero-Malay Population in Indonesia

**DOI:** 10.1155/2019/7629146

**Published:** 2019-03-13

**Authors:** Suriya Loganadan, Murnisari Dardjan, Nani Murniati, Fahmi Oscandar, Yuti Malinda, Dewi Zakiawati

**Affiliations:** ^1^Bachelor of Dental Science, Fakultas Kedokteran Gigi, Universitas Padjadjaran, Bandung, West Java, Indonesia; ^2^Lecturer and Researcher in Oral Biology (Oral Pathology) Department–Forensic Odontology, Fakultas Kedokteran Gigi, Universitas Padjadjaran, Bandung, West Java, Indonesia; ^3^Lecturer and Researcher in Oral Biology (Oral Anatomy) Department–Forensic Odontology, Fakultas Kedokteran Gigi, Universitas Padjadjaran, Bandung, West Java, Indonesia; ^4^Lecturer in Oral and Maxillofacial Radiology Department-Forensic of Dentistry, Universitas Padjadjaran, Bandung, West Java, Indonesia; ^5^Lecturer and Researcher in Oral Biology (Oral Microbiology) Department–Forensic Odontology, Fakultas Kedokteran Gigi, Universitas Padjadjaran, Bandung, West Java, Indonesia; ^6^Lecturer and Researcher in Oral Medicine Department–Forensic Odontology, Fakultas Kedokteran Gigi, Universitas Padjadjaran, Bandung, West Java, Indonesia

## Abstract

**Introduction:**

Human identification is vital not only in legal medicine but also in criminal inquiries and identification. Cheiloscopy is the study of lip prints which are unique, individual, and heritable that is used for personal identification purposes in forensic odontology.

**Objective:**

The aim of this study is to identify the possibility of the child to inherit the lip print patterns from their parents and also to describe the lip print patterns in children and their parents among the Deutero-Malay population.

**Method:**

The descriptive research used lip samples of 90 individuals including father, mother, and a child who are biologically related and their age ranges from 12 to 60 years old. The samples chosen are from the Deutero-Malay ethnic in Indonesia at least for the past two generation who obeys all the exclusion criteria of this research. Purposive nonrandom sampling method was used to collect samples by photography technique using a digital camera, and the data obtained were then analysed using Adobe Photoshop CS3 software. Grooves and wrinkles of primary quadrants one, three, six, and seven of lips were studied according to Suzuki and Tsuchihashi's classification in 1971.

**Result:**

In the present study, it is found that Type I′ (30.28%) is the most dominant lip print pattern and Type I (1.39%) is the least dominant among the Deutero-Malay population. Besides, this study has shown that the similarity of lip print pattern between mother and the child (57.89%) is greater compared to the father and the child (42.22%).

**Conclusion:**

Based on this, we can conclude that lip print can be inherited and dissimilar for every population of race; likewise, the Deutero-Malay population has the Type I′ as the most dominant lip print pattern.

## 1. Introduction

Many scientific studies on lip prints took the attention of many detectives, and it remains as a forensic work of standardized methods to establish the collection and classification of the uniquely patterned grooves, furrows, and wrinkles that comprises the human lip [1–5]. The development of lips starts as early as the fifth week in vitro. During the period of the fifth to sixth week, the components that will become the lower lip begin to form. By the ninth week, the formation of the upper lip will be almost completed and remains unchanged for the rest of a human's life, unless the person undergoes severe trauma or surgery [6] (Table 1).

Furthermore, previous studies have evidence that lip prints are appropriate for the successful identification of a person to crime due to its characteristics such as hereditary, uniqueness and individualistic, permanent, and stability over the time, and it is also influenced by race [7–10]. Therefore, Suzuki and Tsuchihashi proposed a classification system of the lip prints; these are widely used classification in the literature. They classified the natural lip marks or fissures into six types [11].

Furthermore, in Girish R. Dongarwar's study, he has proved that cheiloscopy holds the potential to identify the sex and identity of an individual, as they remain stable over time and unique to the individual, even in the twins and family relatives [9]. Therefore, it is illustrated that there is similarity of lip print pattern among children and parents of every population.

Thus, the aim of this study was to investigate the description of lip print pattern in children and their parents among the Deutero-Malay population.

The objectives of this study are

1. To investigate the description of lip print patterns in children and their parents among Deutero-Malay population
2. To identify the possibility of the child to inherit the lip print patterns from their parents

## 2. Materials and Methods

**Figure 1:**
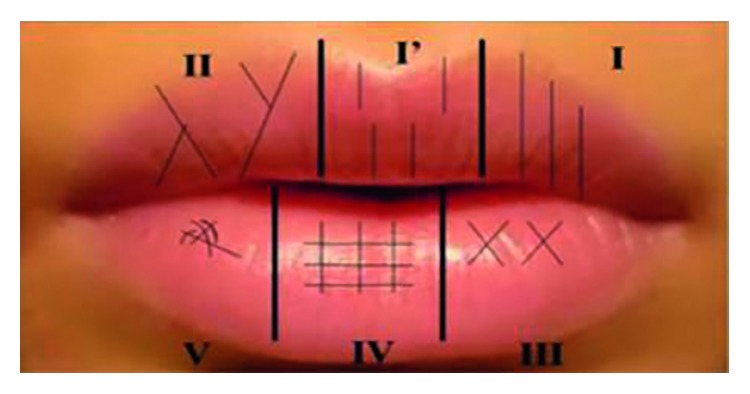
Classification by Suzuki and Tsuchihashi [12].

Ninety samples of the lip which consists of father, mother, and a child (30 families) from the same family, age ranging from 12 to 60 years, were collected from Bandung, Indonesia. The sample size was obtained using Slovin's formula, where it is determined to be 100 pairs of lips with the confidence level of 90% with a margin error of 0.10. Since the data collected for this study are primary data, therefore, there is a drop-out value of 10%. Thus, the total number of samples for this study is as follows: 100–10 = 90 samples of the lip. The Health Research Ethics Committee of Universitas Padjadjaran approved the ethical authorization for this study in December 2017 (REC ref: 1240/UN6. C. 10/PN/2017). The sample was taken using photography technique by a high-clarity camera (DSLR Canon EOS 700D with EFS 18 to 135 mm macro 0.39 m to a 1.3 ft lens with a focal length of 55 mm) as shown in Figure 1. Lux light meter application was used to determine the ideal lighting to take photographs of the sample (56 lx).

**Figure 2:**
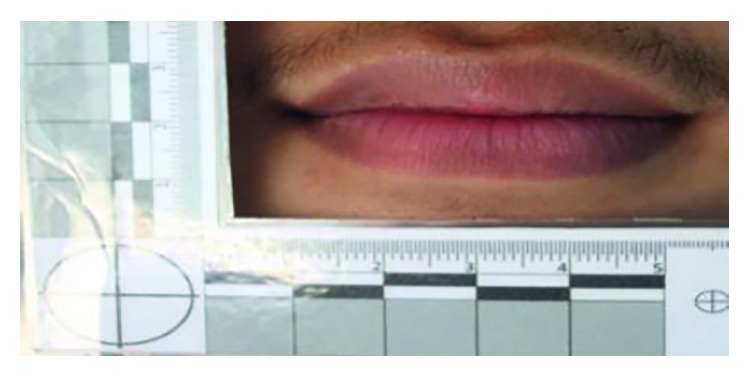
Photography technique of taking lip print [9].

Informed consent was used to get permission before conducting the procedure. A tripod is used to stabilize and elevate the camera so that the pictures taken are clear. Moreover, the ABFO (American Board of Forensic Odontology) scale was used for the subject to hold when the photograph is taken to ensure that the camera is ninety degrees to the plane of the scale when the subject was sitting on a chair. A distance of the camera to the subject's lip was fixed to 0.8 meters, and the distance of the camera lens from the ground was fixed to 1.0 meter. According to Figure 2, using Adobe Photoshop Creative Suite 3 (CS3), the images were zoomed according to preference, and then, the lip sample was divided into four quadrants, and individual colours are drawn for every lip print pattern according to Suzuki and Tsuchihashi's classification using a brush tool [10]. The Suzuki and Tsuchihashi's classification has six types such as Type I (complete straight), Type I′ (partial straight), Type II (branched groove), Type III (intersected groove), Type IV (reticular groove), and Type V (mixed groove) [10].

**Figure 3:**
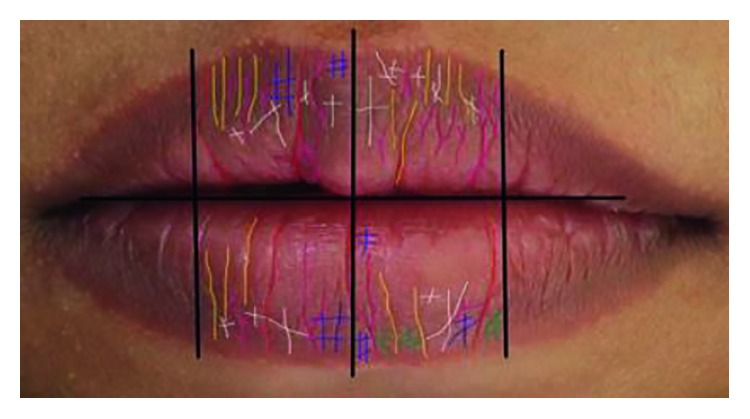
Example of the sample used to analyse the lip print patterns [9].

The samples chosen are from Deutero-Malay ethnic at least for the past two generations, mixed ethnic is not chosen, and biological parents and children were selected. Patients who have any lip abnormalities, congenital disease, inflammation, surgery, trauma, malocclusion, severe crowding, and heavy smokers and who are using fixed orthodontic appliances such as braces were already excluded from the sample to participate in this research (Figure 3).

## 3. Results

To exclude interexaminer variability, all the measurements were prepared by the same examiner using SPSS 20.2, as shown in Tables 2 and 3. To assess the intraexaminer reproducibility, a random sample of 20 photographs of lip print of children and their parents among the Deutero-Malay population in Indonesia is chosen with the mean age of the parents being 52 and the mean age of children being 16.

Lip print patterns of each subject were photographed, and the results obtained were recorded. It was found that every individual has a unique lip print pattern, and there was no individual who shares the same lip print patterns. The division of lip prints and its analysis was in accordance with Suzuki and Tsuchihashi [10].

**Figure 4:**
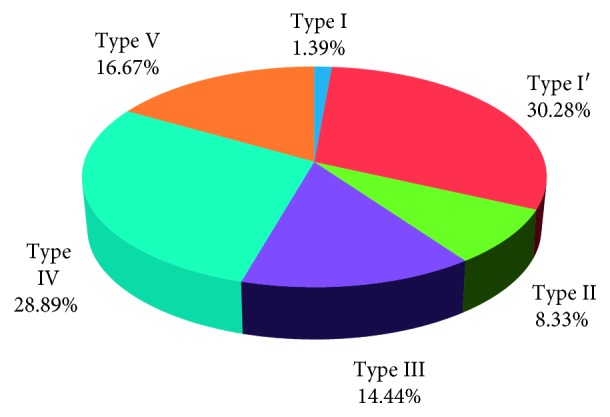
Percentage distribution of lip print patterns among the Deutero-Malay population studied [9].

As shown in Figure 4, in the present study, Type I′ (30.28%) was the most dominant lip print pattern among the Deutero-Malay population, while Type I is the least dominant type with 1.39% in the examined subjects of the Deutero-Malay population.

Based on Table 4, we can see that there is 53.37% resemblance pattern of lip print and 46.67% nonresemblance pattern between father and children. There are 14 children (46.67%) who show category 0 lip print resemblance towards their father whereas 7 (23.33%) children show category 3 lip print resemblance towards their children. Following that, 4 (13.33%) children and 3 (10.00%) children from the sample category 4 and category 1 lip print have resemblance towards their father, respectively. On the other hand, 2 (6.67%) children are grouped into category 2, and none of the children (0.00%) from the sample shows category 5.

Based on Table 5, it can be seen that there is 73.33% of lip print resemblance between mother and children and 26.67% are nonresemblance type. There are 8 (26.67%) children who, respectively, show category 0 and category 4. However, 6 (20.00%) children show category 1 lip print resemblance to their mother. Following that, there are 3 (10.00%) children who, respectively, show category 2 and category 3 lip print resemblance to their mother. However, only 2 (6.67%) children show category 5 from this research.

According to Table 6, it can be seen that the total number of resemblances shown between mother and children is higher compared to that of father and children. This can be seen where the resemblance shown between father and children which is from category 1 to category 5 is only 16 while the total number of resemblance shown between mother and children is 22 which is much higher. Besides, the nonresemblance between father and children is 14 while the nonresemblance between mother and children is only 8. The resemblance of lip print patterns was divided into six categories to identify the resemblance of lip print patterns between children and their parents.  Table 7 shows the total number of resemblance between mother and children is higher compared to father and children. This was concluded when the nonresemblance between father and children was higher in category 0 which means that the child only has one or no same lip print type. In categories four and five where the child has three or all same lip print type on the same segments, was higher between mother and children compared to father and children. Therefore, it can be concluded from Table 7 that children show higher resemblance towards their mother compared to their father.

Table 8 shows the resemblance of lip print pattern between father and children. It was found that there are 42.22% of resemblance and 63.64% of nonresemblance between father and children. Meanwhile, there were 57.89% of resemblance and 36.36% of nonresemblance of lip print patterns between mother and children.

## 4. Discussion

Lip print pattern study requires samples of clear images of lip prints in a standardized position with photography using a digital camera. This research is conducted using samples that fulfilled all the inclusive and exclusive criteria where the total number of the sample used for this sample is 90 which consist of 60 parents (father and mother) and 30 children.

From the result obtained, it can be inferred that a particular lip print is unique and different for every person. This statement can be supported by a research conducted by Jaishankar et al. in the year 2010, where they used 180 individuals which also included pairs of twins to study the pattern of lip prints. Based on their results, they specified that most of the lip print patterns did not comprise of one type of pattern alone but seemed like a mixture of varying types [11–13].

After carrying out this research, it was found that Type I′ (30.28%) is the most dominant lip print type in the population. This also can be supported by a research conducted by Bindal who used 60 samples from the three races of Malaysia and has concluded in the result that Malay population has the most commonly observed pattern as Type I′ (71.25%) [8]. Meanwhile, there is another study proving that Type I′ is most prevalent among North Indian population, and the morphological patterns of lip prints in Mangalorean's by Jeergal et al. in 2016 have also stated that Type I′ is the most dominant among Mangalore population [14].

Furthermore, Type I′ is the most common type of lip print pattern may be due to the presence of Klein zone, which is the centre of lips coated from wrinkles and grooves which can form Type I and Type I′ as the most prevalent lip print pattern [12]. However, on the contrary to this, a study conducted by George et al. in 2016 on 31 Malay families has proven that the most common lip print pattern among the study group is Type I (29.84%) while the rare type is Type V (1.61%) [15].

Besides, in accordance with inheritance, the resemblance of lip print pattern between the parents and children found that children shows higher resemblance towards their mother compared to their father. This finding is very much reasonable because children carry half of the parent's genetic information [16, 17]. However, there is a prospect that children own very minimal similarity of lip print with their parents because of its individuality and uniqueness [14, 15, 18]. Therefore, it can be concluded there are differences and correlations in the results obtained by previous studies as shown in Table 9 compared to this research. Previous studies on lip print patterns that have been done on Deutero-Malay population, include research from countries such as Malaysia, India, and Indonesia, can be a strong evidence that Type I′ is the most prevalent among the Deutero-Malay population similar to some of the previous researches and also as obtained in this research. Furthermore, there are some similarities in basic patterns of lip prints between family members which attributed to the influence of inheritance.

## 5. Conclusion

This study demonstrates the description of lip print patterns in children and their parents among Deutero-Malay population. According to the data obtained, it was revealed that Type I′ (30.28%) is the most dominant lip print pattern and the least is Type I (1.39%). Moreover, the resemblance of lip print pattern between the mother and the child is higher compared to the father and the child. Further studies in similar grounds considering different populations or subethnics and classifications should be done in order to create a comprehensive database, so that the hidden potential of lip prints as an important source of information can be utilized optimally.

As mentioned above, individual identification is an important and challenging task in forensic investigation. Therefore, application of cheiloscopy can be very essential in human identification. The most important characteristics of lip prints are unique and its individuality similar to fingerprint which can be used for criminal findings in the future.

## Figures and Tables

**Table 1:** Suzuki and Tsuchihashi classification of lip print pattern [11].

Classification	Groove
Type I	Complete straight (clear-cut vertical grooves that run across the lips)
Type I′	Partial straight (clear-cut vertical grooves that run across the lips)
Type II	Branched groove (branching Y-shaped pattern)
Type III	Intersected groove (criss-cross grooves)
Type IV	Reticular pattern (grooves that form a rectangular pattern)
Type V	Mixed groove (grooves that do not categorize in any of the above and cannot be differentiated morphologically)

**Table 2:** Pearson correlation for intraobserver.

Subject		Lip print patterns
Children	Pearson correlation sig. (2-tailed)	0.957257
Parents	Pearson correlation sig. (2-tailed)	0.961767

**Table 3:** Pearson correlation for interobserver.

Subject		Lip print patterns
Children	Pearson correlation sig. (2-tailed)	0.965294
Parents	Pearson correlation sig. (2-tailed)	0.981131

**Table 4:** Resemblance of lip print patterns between father and children.

	Category	Total	%
0	Having 1 same lip print type or having no same lip print type	14	46.67
1	Having 2 same lip print types but on different segments	3	10.00
2	Having 3 same lip print types but on different segments	2	6.67
3	Having 2 same lip print types on the same segments	7	23.33
4	Having 3 same lip print types on the same segments	4	13.33
5	Having all same lip print types on the same segments	0	0.00
Total		30	100

**Table 5:** Resemblance of lip print patterns between mother and children.

	Category	Total	%
0	Having 1 same lip print type or having no same lip print type	8	26.67
1	Having 2 same lip print types but on different segments	6	20.00
2	Having 3 same lip print types but on different segments	3	10.00
3	Having 2 same lip print types on the same segments	3	10.00
4	Having 3 same lip print types on the same segments	8	26.67
5	Having all same lip print types on the same segments	2	6.67
Total		30	100.00

**Table 6:** Resemblance of lip print patterns between children and their parents.

	Category	F (C)	M (C)	Total
0	Having 1 same lip print type or having no same lip print type	14	8	22
1	Having 2 same lip print types but on different segments	3	6	9
2	Having 3 same lip print types but on different segments	2	3	5
3	Having 2 same lip print types on the same segments	7	3	10
4	Having 3 same lip print types on the same segments	4	8	12
5	Having all same lip print types on the same segments	0	2	2
Total		30	30	60

**Table 7:** Comparison of resemblance and nonresemblance of lip print patterns between children and their parents.

	Resemblance		Nonresemblance	
Father and children	16	42.11%	14	63.64%
Mother and children	22	57.89%	8	36.36%
Total	38	100%	22	100%

**Table 8:** Resemblance of lip print patterns between children and their parents.

	Category	F (C)	M (C)	Total
0	Having 1 same lip print type or having no same lip print type	14	8	22
1	Having 2 same lip print types but on different segments	3	6	9
2	Having 3 same lip print types but on different segments	2	3	5
3	Having 2 same lip print types on the same segments	7	3	10
4	Having 3 same lip print types on the same segments	4	8	12
5	Having all same lip print types on the same segments	0	2	2
Total		30	30	60

**Table 9:** Sample characteristics of lip print pattern studies.

Population	Researcher	Year	Total no. of samples	Dominant type of lip print pattern
India	Jeergal et al. [14]	2016	200	Type I′
Malaysia	Bindal et al. [8]	2014	60	Type I′
Indonesia	Loganadan [9]	2018	90	Type I′

## Data Availability

The data used to support the findings of this study are available from the corresponding author upon request.
